# Supplementary material for: Cilia locally synthesize proteins to sustain their ultrastructure and functions
Source: Nat Commun. 2021 Nov 30;12:6971. doi: 10.1038/s41467-021-27298-1 (PMC8632896; doi:10.1038/s41467-021-27298-1)
Supplement: Supplementary file 1 — Supplementary Information [file 41467_2021_27298_MOESM1_ESM.pdf]

Supplementary Information

Cilia locally synthesize proteins to sustain their ultrastructure and functions

Kai Hao[1,2], Yawen Chen[1,2], Xiumin Yan[4*], and Xueliang Zhu[1,2,3,*]

[1] State Key Laboratory of Cell Biology, Shanghai Institute of Biochemistry and Cell Biology, Center for Excellence in Molecular Cell Science, Chinese Academy of Sciences, 320 Yueyang Road, Shanghai 200031, China

[2] University of Chinese Academy of Sciences, Beijing 100049, China

[3] School of Life Science, Hangzhou Institute for Advanced Study, University of Chinese Academy of Sciences, Hangzhou 310024, China

[4] Ministry of Education-Shanghai Key Laboratory of Children's Environmental Health, Institute of Early Life Health, Xinhua Hospital, Shanghai Jiao Tong University School of Medicine, Shanghai 200092, China

* These authors jointly supervised this work (e-mail: yanxiumin@xinhuamed.com.cn; xlzhu@sibcb.ac.cn)

Running title: Local protein synthesis in cilia

Supplementary Fig. 1, Hao et al.

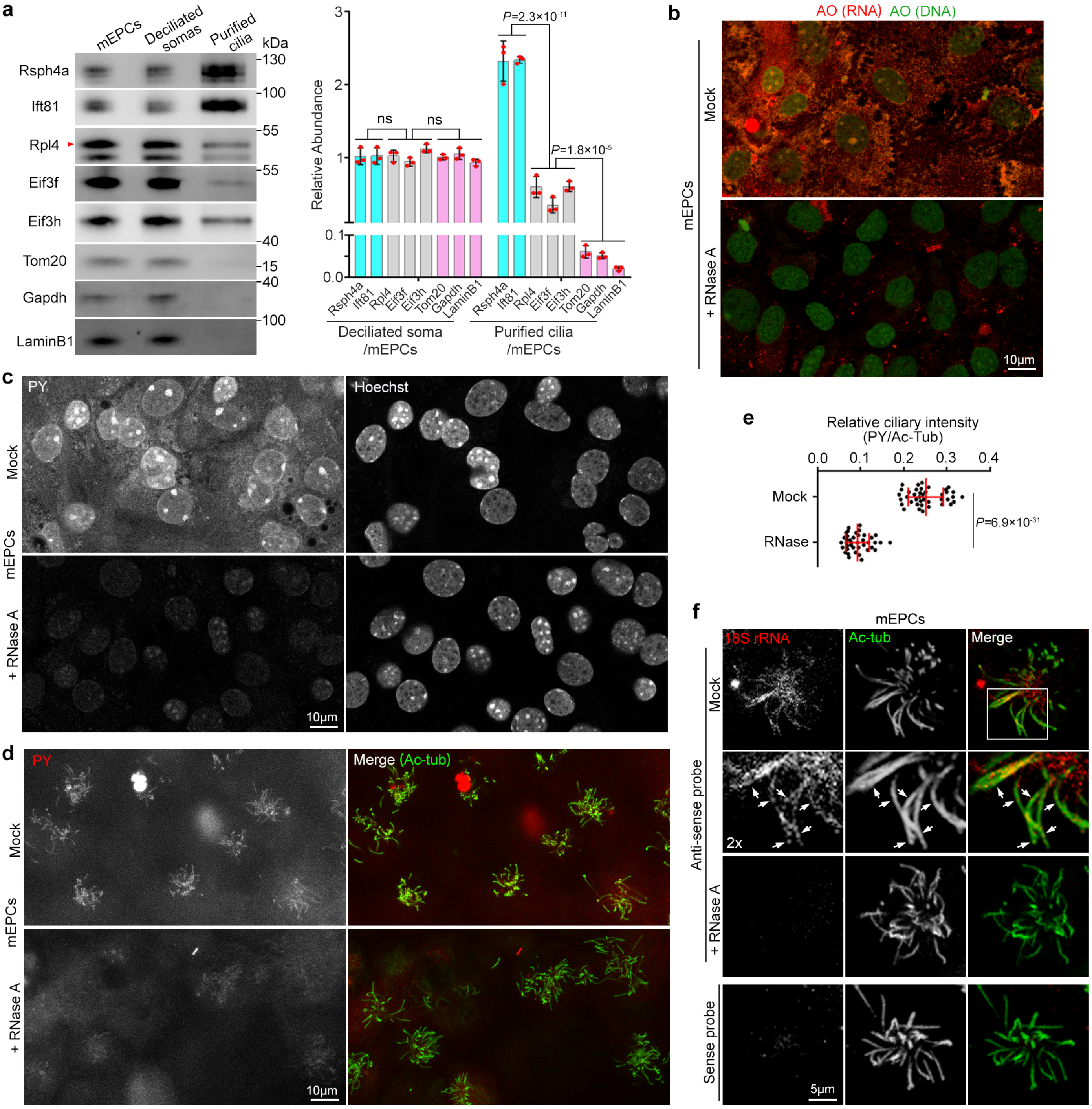

Supplementary Figure 1. Ependymal multicilia contain ribosomal components and RNA (related to Fig. 1).
Day-10 mEPCs were used in the experiments. **(a)** Rpl4, Eif3f, and Eif3h were enriched in purified ependymal multicilia. Ependymal cilia were purified as illustrated in Figure 1a. Equal amount of total proteins from the indicated fractions was loaded in each lane for immunoblotting. Rspha4a and Ift81 served as ciliary markers, whereas Tom20, Gapdh, and LaminB1 were used as markers to assess contaminations from the mitochondrion, cytosol, and nucleus, respectively. Band intensities relative to those of intact mEPCs were measured from three sets of independent immunoblotting results and are presented as mean ± s.d. Two tailed student's t test: ns, no significance. **(b)** Confocal micrographs of AO-staining, imaged at the plane of nuclei. mEPCs were fixed with 4% paraformaldehyde (PFA) at 4℃, treated with just PBS (mock) or 100 μg/ml RNase A in PBS for 30 min at 37℃, and subjected to AO staining. Note that only the red fluorescence for RNA was sensitive to RNase. **(c)** Pyronin Y (PY) preferably recognized RNA. mEPCs were fixed with 4% PFA, permeabilized with 0.5% Triton X-100, and treated PBS or RNase A as in **(b)**. The cells were then stained with PY and Hoest33342, a DNA-specific dye. Confocal imaging was performed at the plane of nuclei. Note that the RNase treatment removed most fluorescent signals, leaving only weak DNA fluorescence in the nuclei. **(d)** Multicilia displayed prominent PY-staining. mEPCs treated as in **(c)** were stained with PY, followed by immunostaining to label Ac-tub. Note that the multiciliary PY signals were sensitive to the RNase treatment. **(e)** Relative fluorescent intensities of ciliary PY. 40 multiciliated mEPCs were measured in each condition. For each cell, total ciliary fluorescent intensity of PY was quantified and normalized to that of Ac-Tub. The bars and errors represent mean + s.d. Two tailed student's t test. **(f)** Ependymal cilia contained 18S rRNA (arrows). mEPCs treated as shown were subjected to FISH to detect 18S rRNA. Ac-tub served as ciliary marker. The framed area was magnified to show details.

Supplementary Fig. 2, Hao et al.

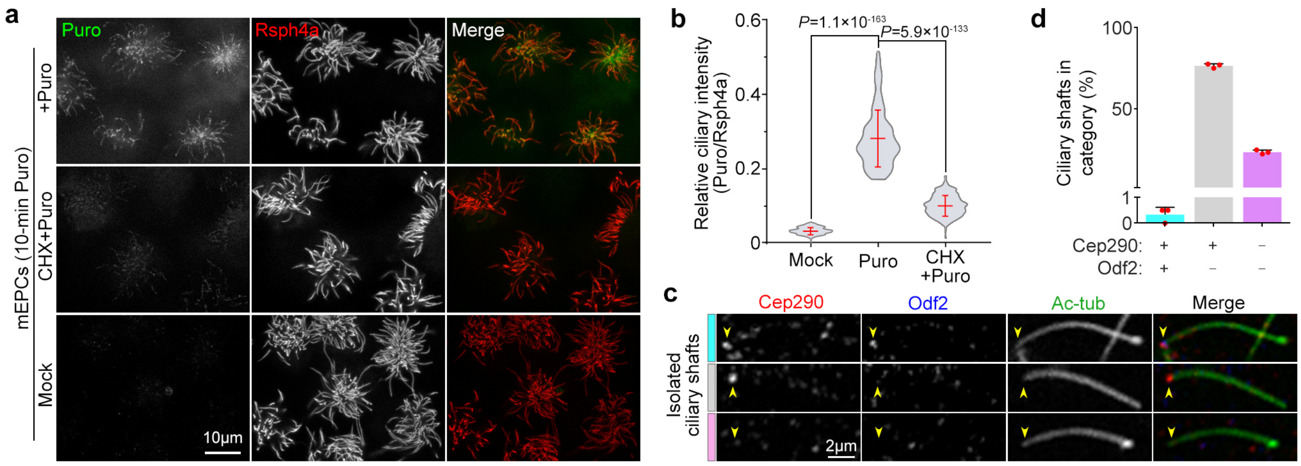

Supplementary Figure 2. Detection of puromycylated peptides in ependymal multicilia and characterization of isolated ciliary shafts (related to Fig. 3).

(a, b) Detection of protein translation-dependent Puro IF signals in multicilia. Day-10 mEPCs were pulse-labeled with Puro for 10 min with or without CHX and then processed for confocal imaging **(a)**. Mock-treated cells served as negative control. Rsph4a served as multi-ciliary marker. Relative IF intensities of ciliary Puro **(b)** were pooled from three independent experiments. 100 multiciliated mEPCs were measured in each experiment and condition. For each cell, the total ciliary IF intensity of Puro was normalized to that of Rsph4a. The bars and errors in the violin plots represent mean ± s.d. Two tailed student's t test. **(c, d)** Characterization of isolated ciliary shafts. Ciliary shafts prepared as illustrated in Figure 3g were immunostained for Ac-tub, Cep290, and Odf2 to visualize ciliary axoneme, TZ, and basal body, respectively **(c)**. Arrowheads point to the ciliary base. Statistical results **(d)**, presented as mean + s.d., were from three independent experiments. 200 ciliary shafts were scored in each experiment and condition.

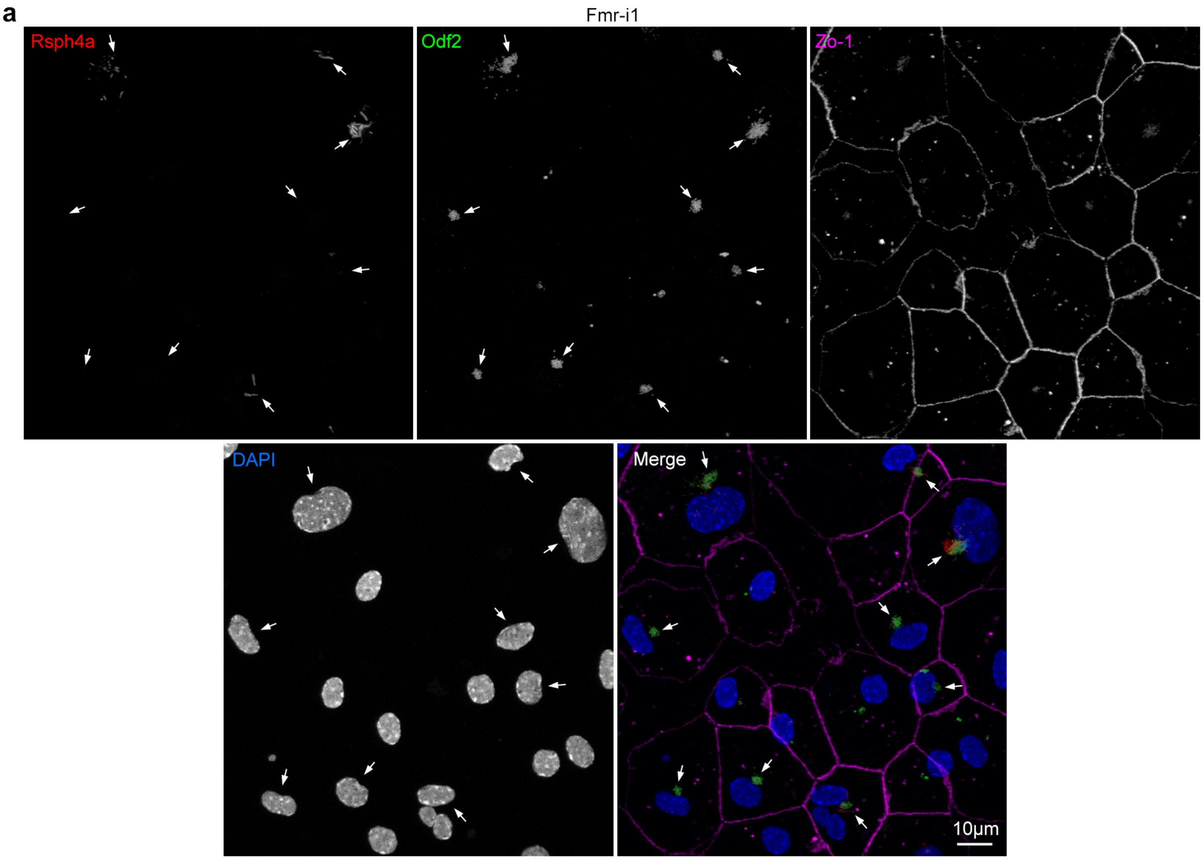

Supplementary Figure 3. Multicilia degeneration in FMRP-depleted mEPCs is not due to cell death (related to Fig. 5). mEPCs transfected with Fmr-i1 as in Figure 5a were fixed at day 12 and immunostained. Rsph4a, Odf2, Zo-1, and DAPI respectively labeled cilia, basal bodies, tight junctions, and nuclei. Arrows point to the basal body cluster and nucleus of multiciliated cells. Note that axonemes were completely lost in several muticiliated cells.

Supplementary Fig. 4, Hao et al.

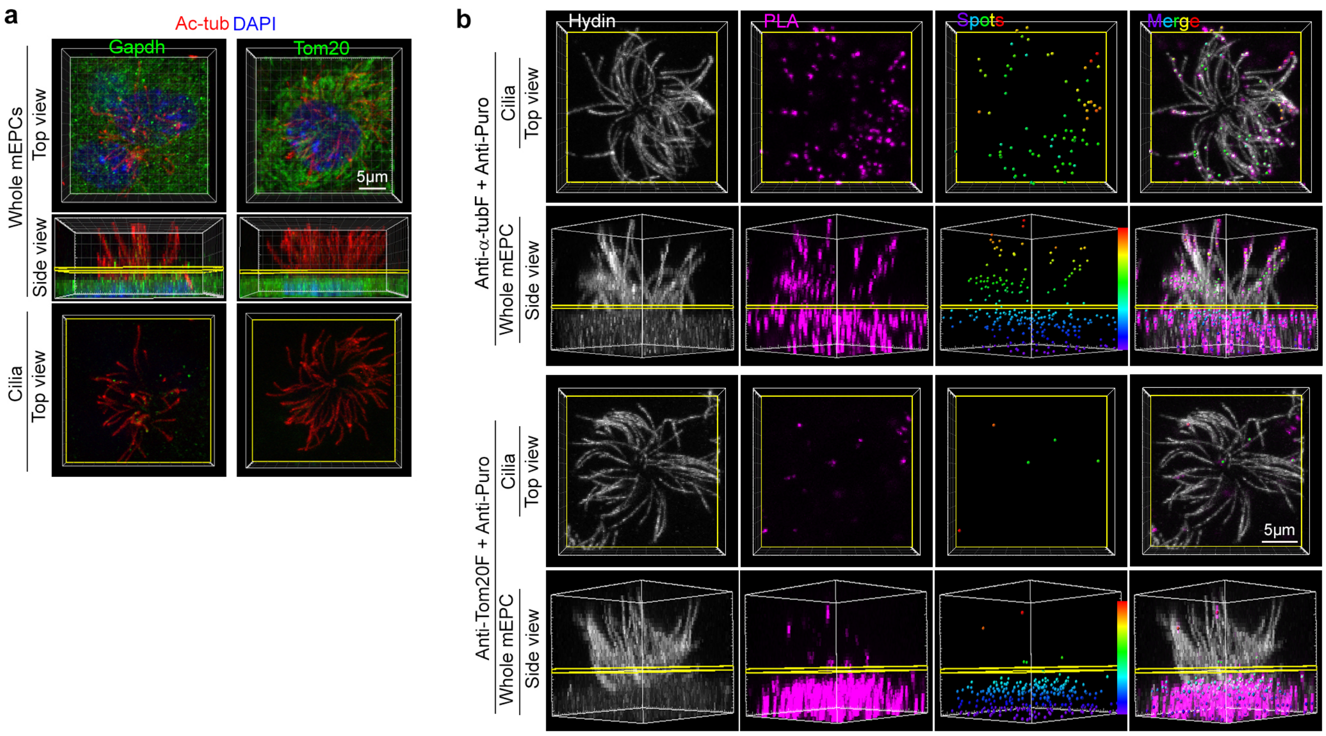

Supplementary Figure 4. Ependymal multicilia locally synthesize α-tubulin but not Tom20 (related to Fig. 8e,f).
(a) Gapdh and Tom20 were rarely detected in multicilia. mEPCs at day 10 were immunostained to visualize Gapdh or Tom20. Ac-tub served as ciliary marker. Nuclear DNA was counterstained with DAPI. The top and middle panels are top and side views of 3D-reconstructed whole mEPCs. The bottom panels show respective top views for the ciliary region (above the yellow planes of the whole mEPCs). **(b)** Representative 3D-reconstructed images showing PLA signals from anti-α-tubF/anti-Puro and anti-Tom20F/anti-Puro pairs, respectively. Puro labeling was performed for 10 min, followed by PLA. Hydin served as ciliary marker. The bottom panels of each set of images show side views of PLA signals, software-generated color-coded PLA spots along the z-axis for statistical analyses[1], and their merged image with the Hydin channel. The corresponding top panels show top views for the ciliary region (above the yellow planes of the whole mEPCs). See Figure 8f for quantification results.

Supplementary Table 1: List of 36 FMRP regulated ciliary mRNA candidates

Gene_ID	Gene Symbol	GO class based on AmiGO 2	Function
ENSMUSG00000000838	Fmr1	ciliary shaft	
ENSMUSG00000072235	Tuba1a	axoneme	α-Tubulin isoform
ENSMUSG00000045136	Tubb2b	axoneme	β-tubulin isoform
ENSMUSG00000062380	Tubb3	axoneme	β-tubulin isoform
ENSMUSG00000062591	Tubb4a	axoneme	β-tubulin isoform
ENSMUST00000069614	Dcdc2a	axoneme	microtubule regulator[2]
ENSMUST00000033642	Dcx	axoneme	microtubule regulator[3]
ENSMUST00000151641	Ttll9	axoneme	tubulin polyglutamylase[4]
ENSMUST00000030951	Ttll10	axoneme	tubulin glycylase[5]
ENSMUSG00000021879	Dnah12	axoneme	dynein arm subunit[6]
ENSMUSG00000042707	Dnali1	axoneme	dynein arm subunit[6]
ENSMUST00000058479	Drc7	axoneme	dynein-docking subunit[7]
ENSMUST00000092684	Ttc25	axoneme	dynein-docking subunit[8]
ENSMUSG00000006464	Bbs1	axoneme	IFT subunit[9]
ENSMUSG00000016637	Ift27	axoneme	IFT subunit[9]
ENSMUSG00000017858	Ift52	axoneme	IFT subunit[9]
ENSMUST00000135973	Armc9	ciliary tip	ciliary stability[10]
ENSMUST00000106227	Cep131	ciliary transition zone	multiciliary assembly[11]
ENSMUST00000059319	Tmem17	ciliary transition zone	Ciliary gate[12]
ENSMUST00000135252	Cfap69	axoneme/motile cilium	flagellum assembly[13]
ENSMUST00000154516	Dnajb13	axoneme	axoneme formation[14]
ENSMUST00000173867	Cct4	cilium	
ENSMUST00000102729	Eps15	ciliary membrane	
ENSMUST00000018711	Gabarap	axoneme	
ENSMUST00000147839	Iqce	ciliary membrane	
ENSMUST00000106407	Rabep2	axoneme	
ENSMUST00000052521	Gas2l2	ciliary basal body	
ENSMUST00000148350	Fbf1	ciliary transition fiber	
ENSMUST00000106591	Agbl4	ciliary basal body	
ENSMUST00000110224	Ttll5	ciliary basal body	
ENSMUST00000048309	Camsap2	ciliary basal body	
ENSMUST00000038842	Ppp1r32	ciliary basal body	
ENSMUST00000028342	Ssna1	ciliary basal body	
ENSMUST00000094156	Fam183b	ciliary basal body	
ENSMUST00000037397	Cep126	ciliary base	
ENSMUST00000021287	Cfap52	ciliary basal body	

Supplementary Table 2: List of primers for cloning

Plasmids	5'-Sequence-3'		Restriction Sites
	Forward Primer	Reverse Primer	
pLV-GFP-RPS3 *(Mus musculus)*	GGACTCAGATCTCGAATGGCGGTGCAGATTTCC	GAAGCTTGAGCTCGATTATGCTGTAGGCACTGGC	Xho I
pLV-GFP-RPL11 *(Mus musculus)*	GGACTCAGATCTCGAATGGCGCAAGATCAAG	GAAGCTTGAGCTCGATTATTTTCCAGGAAGGATG	Xho I
pLV-Centrin1-GFP *(Mus musculus)*	CCGTCAGATCCGCTAGCATGGCGTCCACCTTC	CATGGTGGCGACCGGATAAAGGTTGGTCTTTTTCATGAT	Nhe I/Age I
pcDNA3.0-18s rRNA (490-1489nt) *(Mus musculus)*	TCTAGATGCATGCTCGAGACCCACTCCCGAC	ACCGAGCTCGGATCCTGTTATTGCTCAATCTCGGG	Xho I/ BamH I
pLV-GFP-eIF3d *(Mus musculus)*	GGACTCAGATCTCGAATGGCGAAGTTCATGACA	GAAGCTTGAGCTCGATTAAGTTTCTTCCTCTTCCT	Xho I
pLV-GFP-eIF3h *(Mus musculus)*	GGACTCAGATCTCGAATGGCGTCGCGCAAG	GAAGCTTGAGCTCGATTAATTATTGTATTCTTGAAGAGCC	Xho I
pLV-GFP-eIF3m *(Mus musculus)*	GGACTCAGATCTCGAATGAGCGTCCCGGC	GAAGCTTGAGCTCGATCAGGTATCTGAAAGACTCAAAAGG	Xho I
pLV-GFP-FMRP *(Mus musculus)*	GGACTCAGATCTCGAATGGAGGAGCTGGTGGTGGA	GAAGCTTGAGCTCGATTAGGGTACTCCATTCACCAGCG	Xho I
pLV-GFP-mtFMRP *(Mus musculus)*	CTCAAGCTTCGAATTCGTTCCAGCGGTAGAAATCACCCAC	GTCGACTGCAGAATTTCAAGACCGTGGCAGGAGCT	EcoR I
pYr1.1-GFP-FMRP *(Mus musculus)*	GGTGGCGCTACCGGTATGGTGAGCAAGGGC	ATCTAGATCCGGTGGATCTCGAGTTAGGGTACTCCATTCACCA	Age I/Xho I
pYr1.1-GFP-mtFMRP *(Mus musculus)*	GGTGGCGCTACCGGTATGGTGAGCAAGGGC	ATCTAGATCCGGTGGATCTCGAGTCAAGACCGTGGCAG	Age I/Xho I
pYr1.1-Centrin1-GFP *(Mus musculus)*	AACCGTCAGATCCGCTAGCATGGCGTCCACCTTC	ATCTAGATCCGGTGGATCTCGAGTTACTTGTACAGCTCGTCC	Nhe I/Xho I

Supplementary Table 3: List of antibodies for immunoblotting (IB) and immunofluorescence (IF)

Primary antibodies					
Antigen	Species	Supplier	Catalog	Dilute	
				IB	IF
Rsph4a	rabbit	home-made		1:1000	1:400
IFT81	rabbit	Proteintech	11744-1-AP	1:1000	
RPL4	rabbit	Proteintech	11302-1-AP	1:1000	
eIF3d	mouse	Santa Cruz	sc-271516	1:1000	
eIF3f	rabbit	Bethyl	A303-005A	1:1000	1:200
eIF3h	rabbit	Cell signaling	3413	1:1000	1:200
Tom20	rabbit	Proteintech	11802-1-AP	1:1000	1:200
Gapdh	rabbit	Abcam	ab181603	1:1000	1:200
LaminB1	rabbit	Proteintech	12987-1-AP	1:1000	
RPL10A	rabbit	Proteintech	16681-1-AP		1:200
RPL11	rabbit	Proteintech	16277-1-AP		1:200
RPS3	rabbit	Proteintech	11990-1-AP		1:200
Acetylated Tubulin	mouse	Sigma-Aldrich	T6793	1:1000	1:1000
eIF3b	goat	Santa Cruz	sc-16377		1:200
eIF3m	rabbit	home-made			1:200
eIF4E	rabbit	Cell signaling	2067		1:200
eIF4G	rabbit	Cell signaling	2498		1:200
Puromycin	mouse	Merk Millipore	MABE343		1:200
Digoxigenin	sheep	Roche	11333089001		1:200
Odf2	guinea pig	home-made			1:200
Cep290	rabbit	home-made			1:200
FMRP	rabbit	Abcam	ab17722	1:1000	1:1000
Hydin	guinea pig	home-made			1:200
Cep164	rabbit	home-made			1:200
Zo-1	mouse	ThermoFisher	33-9100		1:1000
GFP	rabbit	MBL International	598	1:1000	
α-tubulin	rabbit	Proteintech	11224-1-AP		1:800
α-tubulin(C-terminal)	rabbit	Abcam	ab15246		1:100
β-Tubulin	rabbit	Abcam	ab155311		1:200
**Secondary antibodies**					
**Name**	**Conjugates**	**Species**	**Supplier**	**Catalog**	**Dilute**
anti-Mouse IgG (H+L)	HRP	goat	ThermoFisher	G-21040	1:1000
anti-Rabbit IgG (H+L)	HRP	goat	ThermoFisher	G-21234	1:1000
anti-Mouse IgG (H+L)	Alexa Fluor 405	goat	ThermoFisher	A-31553	1:500

anti-Mouse IgG (H+L)	Alexa Fluor 488	donkey	ThermoFisher	A-21202	1:1000
anti-Rabbit IgG (H+L)	Alexa Fluor 488	donkey	ThermoFisher	A-21206	1:1000
anti-Guinea Pig IgG (H+L)	Alexa Fluor 488	donkey	Jackson ImmunoResearch	706-545-148	1:500
anti-Rabbit IgG (H+L)	Cy3	donkey	Jackson ImmunoResearch	711-165-152	1:1000
anti-Guinea Pig IgG (H+L)	Cy3	donkey	Jackson ImmunoResearch	706-165-148	1:1000
anti-Goat IgG (H+L)	Alexa Fluor 546	donkey	ThermoFisher	A-11056	1:1000
anti-Sheep IgG (H+L)	Alexa Fluor 546	donkey	ThermoFisher	A-21098	1:1000
anti-Mouse IgG (H+L)	Alexa Fluor 647	donkey	ThermoFisher	A-31571	1:1000
anti-Rabbit IgG (H+L)	Alexa Fluor 647	goat	ThermoFisher	A-21245	1:1000
anti-Guinea Pig IgG (H+L)	Alexa Fluor 647	donkey	Jackson ImmunoResearch	706-605-148	1:1000
anti-Mouse IgG (H+L)	PLA^{minus} probe	donkey	Sigma-Aldrich	DUO92004	1:5
anti-Rabbit IgG (H+L)	PLA^{plus} probe	donkey	Sigma-Aldrich	DUO92002	1:5

Supplementary Table 4: List of probes for smFISH

Ribo α-Tubulin smFISH Probe Mix		
Target	CDS of Tuba1a, Tuba1b, Tuba1c	
Fluorophore	Cy3	
5'-Sequence-3'	AATGGTCTTGTCACTTGGCA	ATGTCGAGGTTTCTACGACA
	GTTGAAGGAGTCATCTCCTC	CCATCAAATCTGAGGGAAGC
	CTCCTGTCTCACTGAAGAAG	TGTCAGATCAACATTCAGGG
	TTCCAGGTCTACGAACACTG	AGATGACAGGGGCATAAGTG
	GAACTTCATCGATGACCGTG	ATGTATTTACCATGGCGAGG
	CGAATCCTGTCCAGGACAAG	CAGCATTGACATCTTTGGGA
	GAAAACCAAGAAGCCCTGGA	CAGCAATGGCTGTGGTGTTG
	GGGCTGGGTAAATGGAGAAC	AACTTGTGATCTAGGCGAGC
	AGGATGGAATTGTAGGGCTC	ACGCTTGGCATACATCAGAT
	AAGGCACAATCAGAGTGCTC	CCACATACCAGTGCACAAAG
Ribo β-Tubulin smFISH Probe Mix		
Target	CDS of Tubb2a, Tubb2b	
Fluorophore	Cy3	
5'-Sequence-3'	CTGATCTTGCTGATGAGCAG	ACATGCGGCCACGGAAAATG
	TTCATGATGCGGTCTGGGTA	AAGTAGCTGCTGTTCTTGTT
	TGAGGGCATGACGCTGAAGG	TCTTGACGTTGTTGGGGATC
	ACTGAGAGGGTGGCATTATA	CGAGGAGGGATGTCACACAC
	TGACACCAGGTGGTTGAGAT	ATGAAGGTGGCTGACATCTT
	GAATGGCACCATGTTCACGG	GAGATGCGCTTGAACAGCTC
	TGGCATGAAGAAGTGCAGGC	CGTGTACCAGTGCAGGAAAG
	GGCTGGTCAGAGGTGCAAAG	AGGTCATTCATGTTGCTCTC
	TCGAACATCTGCTGGGTCAG	GTACTGCTGGTACTCAGACA
	AGGCAGCCATCATGTTCTTG	AGTGAGTGGGTCAGCTGGAA

Supplementary Reference

1. Cioni JM*, et al.* Late Endosomes Act as mRNA Translation Platforms and Sustain Mitochondria in Axons. *Cell* **176**, 56-72 e15 (2019).

2. Grati M*, et al.* A missense mutation in DCDC2 causes human recessive deafness DFNB66, likely by interfering with sensory hair cell and supporting cell cilia length regulation. *Hum Mol Genet* **24**, 2482-2491 (2015).

3. Moores CA, Perderiset M, Francis F, Chelly J, Houdusse A, Milligan RA. Mechanism of microtubule stabilization by doublecortin. *Mol Cell* **14**, 833-839 (2004).

4. Konno A*, et al.* Ttll9-/- mice sperm flagella show shortening of doublet 7, reduction of doublet 5 polyglutamylation and a stall in beating. *J Cell Sci* **129**, 2757-2766 (2016).

5. Ikegami K, Setou M. TTLL10 can perform tubulin glycylation when co-expressed with TTLL8. *FEBS Lett* **583**, 1957-1963 (2009).

6. King SM. Axonemal Dynein Arms. *Cold Spring Harb Perspect Biol* **8**, a028100 (2016).

7. Morohoshi A*, et al.* Nexin-Dynein regulatory complex component DRC7 but not FBXL13 is required for sperm flagellum formation and male fertility in mice. *PLoS Genet* **16**, e1008585 (2020).

8. Wallmeier J*, et al.* TTC25 Deficiency Results in Defects of the Outer Dynein Arm Docking Machinery and Primary Ciliary Dyskinesia with Left-Right Body Asymmetry Randomization. *Am J Hum Genet* **99**, 460-469 (2016).

9. Ishikawa H, Marshall WF. Intraflagellar Transport and Ciliary Dynamics. *Cold Spring Harb Perspect Biol* **9**, a021998 (2017).

10. Latour BL*, et al.* Dysfunction of the ciliary ARMC9/TOGARAM1 protein module causes Joubert syndrome. *J Clin Invest* **130**, 4423-4439 (2020).

11. Zhao H*, et al.* Fibrogranular materials function as organizers to ensure the fidelity of multiciliary assembly. *Nat Commun* **12**, 1273 (2021).

12. Garcia-Gonzalo FR, Reiter JF. Open Sesame: How Transition Fibers and the Transition Zone Control Ciliary Composition. *Cold Spring Harb Perspect Biol* **9**, a028134 (2017).

13. Dong FN*, et al.* Absence of CFAP69 Causes Male Infertility due to Multiple Morphological Abnormalities of the Flagella in Human and Mouse. *Am J Hum Genet* **102**, 636-648 (2018).

14. El Khouri E*, et al.* Mutations in DNAJB13, Encoding an HSP40 Family Member, Cause Primary Ciliary Dyskinesia and Male Infertility. *Am J Hum Genet* **99**, 489-500 (2016).
