# Supplementary material for: Cilia locally synthesize proteins to sustain their ultrastructure and functions
Source: Nat Commun. 2021 Nov 30;12:6971. doi: 10.1038/s41467-021-27298-1 (PMC8632896; doi:10.1038/s41467-021-27298-1)
Supplement: Supplementary file 3 — Description of Additional Supplementary Files [file 41467_2021_27298_MOESM3_ESM.pdf]

Description of Additional Supplementary Files

File Name: Supplementary Data 1
Description: RNA deep sequencing results of multicilia purified respectively from Ctrl-i- and Fmr-i1-treated mEPC

File Name: Supplementary Movie 1
Description: Multicilia degeneration of Fmr-i1-treated mEPCs (related to Fig. 5f). mEPCs were treated and live imaged as described in Figure 5e. z-stack images of representative cells at each time point were 2D projected and assembled into a time-lapse movie playing back at 12 frames per sec (fps). Time stamps are in the format of hours: minutes. Centrin1-GFP and siR-tub labeled basal bodies and ciliary axonemes, respectively. Representative frames are shown in Figure 5f.

File Name: Supplementary Movie 2
Description: Distribution of punctate PLA fluorescent signals (red) of puromycylated -tubulin peptides along multicilia (blue) (related to Figure 8c). Day-10 mEPCs were subjected to PLA with anti-- tubF and anti-Puro antibodies and imaged at 0.5-µm intervals. Hydin (blue) served as ciliary marker. The ciliary region of a representative cell was 3D-reconstructed by using Imaris (Bitplane). 2D-projected images of the same cell is also shown in Figure 8c.
