# Supplementary material for: Cilia locally synthesize proteins to sustain their ultrastructure and functions
Source: Nat Commun. 2021 Nov 30;12:6971. doi: 10.1038/s41467-021-27298-1 (PMC8632896; doi:10.1038/s41467-021-27298-1)
Supplement: Supplementary file 7 — Reporting Summary [file 41467_2021_27298_MOESM7_ESM.pdf]

nature research

Corresponding author(s): Xueliang Zhu, Xiumin Yan

Last updated by author(s): Oct 28, 2021

Reporting Summary

Nature Research wishes to improve the reproducibility of the work that we publish. This form provides structure for consistency and transparency in reporting. For further information on Nature Research policies, see our Editorial Policies and the Editorial Policy Checklist.

Statistics

For all statistical analyses, confirm that the following items are present in the figure legend, table legend, main text, or Methods section.

n/a	Confirmed	
☐	☒	The exact sample size (n) for each experimental group/condition, given as a discrete number and unit of measurement
☐	☒	A statement on whether measurements were taken from distinct samples or whether the same sample was measured repeatedly
☐	☒	The statistical test(s) used AND whether they are one- or two-sided<br>*Only common tests should be described solely by name; describe more complex techniques in the Methods section.*
☐	☒	A description of all covariates tested
☐	☒	A description of any assumptions or corrections, such as tests of normality and adjustment for multiple comparisons
☐	☒	A full description of the statistical parameters including central tendency (e.g. means) or other basic estimates (e.g. regression coefficient) AND variation (e.g. standard deviation) or associated estimates of uncertainty (e.g. confidence intervals)
☐	☒	For null hypothesis testing, the test statistic (e.g. F, t, r) with confidence intervals, effect sizes, degrees of freedom and P value noted<br>*Give P values as exact values whenever suitable.*
☒	☐	For Bayesian analysis, information on the choice of priors and Markov chain Monte Carlo settings
☒	☐	For hierarchical and complex designs, identification of the appropriate level for tests and full reporting of outcomes
☒	☐	Estimates of effect sizes (e.g. Cohen's d, Pearson's r), indicating how they were calculated

Our web collection on statistics for biologists contains articles on many of the points above.

Software and code

Policy information about availability of computer code

Data collection	Images were collected on Leica TCS SP8 WLL system, GE DeltaVision OMX SR Imaging system and Olympus SpinSR10 spinning disk system.
Data analysis	Data were analyzed using Adobe photoshop(Creative Cloud,2015), Lecia Application Suite X(LAS X), Fiji(Automatic updated), Bitplane Imaris(Version 7.6.5), cellSens Dimension, SoftWoRX(Version 7.0) and Graphpad Prism(Version 8.3.0). The cleaned reads were mapped to the mouse reference genome using Hist (Version 2.0.1). The FPKM values were calculated by Cuffdiff (Version 2.2.1).

For manuscripts utilizing custom algorithms or software that are central to the research but not yet described in published literature, software must be made available to editors and reviewers. We strongly encourage code deposition in a community repository (e.g. GitHub). See the Nature Research guidelines for submitting code & software for further information.

Data

Policy information about availability of data

All manuscripts must include a data availability statement. This statement should provide the following information, where applicable:

- Accession codes, unique identifiers, or web links for publicly available datasets
- A list of figures that have associated raw data
- A description of any restrictions on data availability

The Ciliary RNA-Seq datasets generated and analyzed during the current study have been deposited in the GEO (Gene Expression Omnibus) with the accession codes: GSE179935 [https://www.ncbi.nlm.nih.gov/geo/query/acc.cgi?acc=GSE179935]. Raw data of proteomic analysis results (Fig. 1b and 4a) have been deposited to the ProteomeXchange Consortium via the iProX partner repository with the accession code (PXD029174) [https://www.iprox.cn//page/SCV017.html?query=PXD029174]. Fig 7c, Source data, including uncropped immunoblots, are provided with this paper.

Field-specific reporting

Please select the one below that is the best fit for your research. If you are not sure, read the appropriate sections before making your selection.

☒ Life sciences ☐ Behavioural & social sciences ☐ Ecological, evolutionary & environmental sciences

For a reference copy of the document with all sections, see nature.com/documents/nr-reporting-summary-flat.pdf

Life sciences study design

All studies must disclose on these points even when the disclosure is negative.

Sample size	Sample sizes were not predetermined with any statistical method and they were chosen dependent on availability and general guideline to meet requirements for statistical analyses.
Data exclusions	No data were excluded for the analyses.
Replication	Reproducibility was confirmed. The number of independent experiments is described in the figure legends.
Randomization	In our experimental design, we ensured that similar numbers of cells were randomly assigned to each group and subjected to each treatment that we tested. We imaged cells from multiple randomly-chosen fields in each experiment. For statistics, all cells in the chosen fields were scored.
Blinding	To avoid mistake, we clearly marked all the image data for all the samples in our experiments. Therefore, no blinding has been used in this study.

Reporting for specific materials, systems and methods

We require information from authors about some types of materials, experimental systems and methods used in many studies. Here, indicate whether each material, system or method listed is relevant to your study. If you are not sure if a list item applies to your research, read the appropriate section before selecting a response.

Materials & experimental systems

n/a	Involved in the study	
☐	☒	Antibodies
☐	☒	Eukaryotic cell lines
☒	☐	Palaeontology and archaeology
☐	☒	Animals and other organisms
☒	☐	Human research participants
☒	☐	Clinical data
☒	☐	Dual use research of concern

Methods

n/a	Involved in the study	
☒	☐	ChIP-seq
☒	☐	Flow cytometry
☒	☐	MRI-based neuroimaging

Antibodies

Antibodies used

The source, identifier and dilution information of all antibodies used in this work were listed in supplementary Table 3.
Commercial Primary antibodies:
Antigen Species Supplier Cat. # IB IF
IFT81 rabbit Proteintech 11744-1-AP 1:1000
RPL4 rabbit Proteintech 11302-1-AP 1:1000
eIF3d mouse Santa Cruz sc-271516 1:1000
eIF3f rabbit Bethyl A303-005A 1:1000 1:200
eIF3h rabbit Cell signaling 3413 1:1000 1:200
Tom20 rabbit Proteintech 11802-1-AP 1:1000 1:200
Gapdh rabbit Abcam ab181603 1:1000 1:200
LaminB1 rabbit Proteintech 12987-1-AP 1:1000
RPL10A rabbit Proteintech 16681-1-AP 1:200
RPL11 rabbit Proteintech 16277-1-AP 1:200
RPS3 rabbit Proteintech 11990-1-AP 1:200
Acetylated Tubulin mouse Sigma-Aldrich T6793 1:1000 1:1000
eIF3b goat Santa Cruz sc-16377 1:200
eIF4E rabbit Cell signaling 2067 1:200
eIF4G rabbit Cell signaling 2498 1:200
Puromycin mouse Merk Millipore MABE343 1:200
FMRP rabbit Abcam ab17722 1:1000 1:1000
Zo-1 mouse ThermoFisher 33-9100 1:1000

GFP rabbit MBL International 598 1:1000
α-tubulin rabbit Proteintech 11224-1-AP 1:800
α-tubulin(C-terminal) rabbit Abcam ab15246 1:100
β-Tubulin rabbit Abcam ab155311 1:200
Home-made primary antibodies:
Rsph4a rabbit 1:1000(IB) 1:400 (IF)
eIF3m rabbit 1:200 (IF)
Odf2 guinea pig 1:200 (IF)
Cep290 rabbit 1:200 (IF)
Cep164 rabbit 1:200 (IF)
Hydin guinea pig 1:200 (IF)
Secondary antibodies:

Name	Supplier	Cat. #	Dilute
goat anti-Mouse IgG (H+L) HRP	ThermoFisher	G-21040	1:1000
goat anti-Rabbit IgG (H+L) HRP	ThermoFisher	G-21234	1:1000
goat anti-Mouse IgG (H+L) Alexa Fluor 405	ThermoFisher	A-31553	1:500
donkeyanti-Mouse IgG (H+L) Alexa Fluor 488	ThermoFisher	A-21202	1:1000
donkeyanti-Rabbit IgG (H+L) Alexa Fluor 488	ThermoFisher	A-21206	1:1000
donkey anti-Guinea Pig IgG (H+L) Alexa Fluor 488	Jackson ImmunoResearch	706-545-148	1:500
donkey anti-Rabbit IgG (H+L) Cy3	Jackson ImmunoResearch	711-165-152	1:1000
donkey anti-Guinea Pig IgG (H+L) Cy3	Jackson ImmunoResearch	706-165-148	1:1000
donkey anti-Goat IgG (H+L) Alexa Fluor 546	ThermoFisher	A-11056	1:1000
donkey anti-Mouse IgG (H+L) Alexa Fluor 647	ThermoFisher	A-31571	1:1000
goat anti-Rabbit IgG (H+L) Alexa Fluor 647	ThermoFisher	A-21245	1:1000
donkey anti-Guinea Pig IgG (H+L) Alexa Fluor 647	Jackson ImmunoResearch	706-605-148	1:1000
donkey anti-Mouse IgG (H+L) PLAminus probe	Sigma-Aldrich	DUO92004	1:5
donkey anti-Rabbit IgG (H+L) PLAminus plus	Sigma-Aldrich	DUO92002	1:5

Validation

All commercially available primary antibodies have been validated by the manufactures.
IFT81 (https://www.ptglab.com/products/IFT81-Antibody-11744-1-AP.htm);
RPL4 (https://www.ptglab.com/products/RPL4-Antibody-11302-1-AP.htm);
eIF3d(https://www.scbt.com/zh/p/eif3zeta-antibody-a-3?requestFrom=search)
eIF3f (https://www.bethyl.com/product/A303-005A/eIF3F+Antibody);
eIF3H (https://www.cellsignal.com/products/primary-antibodies/eif3h-d9c1-xp-rabbit-mab/3413);
Tom20 (https://www.ptglab.com/products/TOM20-Antibody-11802-1-AP.htm);
Gapdh (https://www.abcam.cn/gapdh-antibody-epr16884-loading-control-ab181603.html）；
LaminB1 (https://www.ptglab.com/products/LMNB1-Antibody-12987-1-AP.htm);
RPL10A (https://www.ptglab.com/products/RPL10A-Antibody-16681-1-AP.htm);
RPL11 (https://www.ptglab.com/products/RPL11-Antibody-16277-1-AP.htm);
RPS3 (https://www.ptglab.com/products/RPS3-Antibody-11990-1-AP.htm);
Acetylated Tubulin (https://www.sigmaaldrich.cn/CN/en/product/sigma/t6793?context=product);
eIF3b (https://www.scbt.com/p/eif3eta-antibody-n-20/);
eIF4E(https://www.cellsignal.com/products/primary-antibodies/eif4e-c46h6-rabbit-mab/2067);
eIF4G(https://www.cellsignal.com/products/primary-antibodies/eif4g-antibody/2498);
Puromycin (https://www.merckmillipore.com/CN/en/product/Anti-Puromycin-Antibody-clone-12D10,MM_NF-MABE343);
FMRP (https://www.abcam.com/fmrp-antibody-ab17722.html);
Zo-1 (https://www.thermofisher.cn/cn/zh/antibody/product/ZO-1-Antibody-clone-ZO1-1A12-Monoclonal/33-9100);
GFP (https://www.mblbio.com/bio/g/dtl/A/?pcd=598)'
α-tubulin （https://www.ptglab.com/products/TUBA1B-Antibody-11224-1-AP.htm);
α-tubulin(C-terminal) (https://www.abcam.cn/alpha-tubulin-antibody-microtubule-marker-ab15246.html);
β-Tubulin (https://www.abcam.cn/tubb2a--tubb2b-antibody-ab155311.html)
Home-made rabbit anti-Rsph4a, rabbit anti-eIF3m, guinea pig anti-Odf2, rabbit anti-cep290, guinea pig anti-Hydin, rabbit anti-Cep164 have been validated in previously published papers.
Rabbit anti-Rsph4a and guinea pig anti-Hydin (Liu et al., 2021. Wdr47, Camsaps, and Katanin cooperate to generate ciliary central microtubules. Nat Commun 4;12(1):5796.doi: 10.1038/s41467-021-26058-5)
Rabbit anti-Cep290 (Duan et al. 2021. Rabl2 GTP hydrolysis licenses BBSome-mediated export to fine-tune ciliary signaling. EMBO J 40:e105499.)
Rabbit anti-eIF3m (Zeng et al., 2013. The m subunit of murine translation initiation factor eIF3 maintains the integrity of the eIF3 complex and is required for embryonic development, homeostasis, and organ size control.J Biol Chem. Oct 18;288(42):30087-30093. doi: 10.1074/jbc.M113.506147)
Rabbit anti-Cep164 and guinea pig anti-Odf2 (Zhao et al., 2021. Fibrogranular materials function as organizers to ensure the fidelity of multiciliary assembly . Nat Commun. Feb 24;12(1):1273.doi: 10.1038/s41467-021-21506-8.)

Eukaryotic cell lines

Policy information about cell lines

Cell line source(s)

HEK293T (ATCC), HEK293A (Thermo Fisher)

Authentication

The cell lines have been authenticated by STR profiling by the vendor. No further authentication was performed.

Mycoplasma contamination	All cell lines tested negative for mycoplasma contamination.
Commonly misidentified lines (See ICLAC register)	No commonly misidentified cell lines were used.

Animals and other organisms

Policy information about studies involving animals; ARRIVE guidelines recommended for reporting animal research

Laboratory animals	Postnatal day 0 mouse pups of either sex from C57BL/6J strain used for primary cell culture.
Wild animals	No wild animals were used.
Field-collected samples	No field-collected samples were used.
Ethics oversight	Experiments involving mouse tissues were performed in accordance with protocols approved by the Institutional Animal Care and Use Committee of CAS Center for Excellence in molecular Cell Science, Shanghai Institute of Biochemistry and Cell Biology, Chinese academy of Sciences.

Note that full information on the approval of the study protocol must also be provided in the manuscript.
